# Supplementary material for: Introducing the municipal digital offering index for evaluating online services and addressing the digital divide
Source: PeerJ Comput Sci. 2025 Sep 3;11:e3049. doi: 10.7717/peerj-cs.3049 (PMC12453795; doi:10.7717/peerj-cs.3049)
Supplement: Supplemental Information 1 [file peerj-cs-11-3049-s001.docx]

**Table A1: Dimensions’ definitions and subdimensions considered in the research instrument.**

| **Dimension 1: Municipal Data - Information from external sources**  This dimension encompasses the collection and presentation of various statistical and demographic characteristics of Chilean municipalities, as provided by government sources. It focuses on gathering comprehensive information regarding the general attributes and socio-economic indicators of each municipality. |
| --- |
| Municipality, Region, Community Development Index (CDI) 2020; Size; Percentage of Urban Population, Population Quantity, Poverty Rate %, Population Density, Education (Proportions without schooling, with basic schooling, intermediate schooling, and with higher education), Municipal Personnel Expenses (in Million $); Total Municipal Expenses (Accrued in Thousand $); Income from Municipal Patents (in Million $); Income from Municipal Circulation Permits (in Million $) |
| **Dimension 2: Community Information Hub**  This dimension refers to the type and quality of one-way online information provided by the municipality to the community. It encompasses various categories of information aimed at informing, educating, and engaging residents. |
| - Information: Relevant data and resources for the community, including FAQs and incident reports. - Benefits: Free services aimed at supporting community members, such as scholarships and grants. - Municipal Information: Background information about the municipal institution, including its vision and mission. - Municipal Structure: Details about the organization of the municipality, such as the organizational chart and information about the mayor. - COSOC (Communal Council for Civil Society Organizations): A platform fostering collaboration between associations and citizen participation. - Culture and Recreation: Information about cultural and recreational activities organized by the municipality. - Education: Details about educational support provided by the municipality within the community. - Health: Information concerning community health centers or agreements related to healthcare services. - Environment: Spaces and initiatives dedicated to environmental conservation or green recreational areas. - Municipal Units: Indication of official institution addresses and other relevant centers within the community. - Projects: Overview of projects initiated by the municipality for the community's benefit. - Transparency Law: Specifics regarding compliance with transparency laws within the municipality. - Lobbying Law: Exclusive platform containing information required by lobbying laws. |
| **Dimension 3: Bidirectional Transactions**  This dimension assesses the level of interaction and transactional capabilities offered by the website to facilitate user engagement. It evaluates the extent to which the website enables users to carry out digital procedures efficiently and access dynamic information. |
| - Online Processing: Digital processing capabilities accessible on the website. - Information on Procedures: Details about procedures conducted within the municipality. - Online Signature: Facility for electronic signatures provided on the website. - Interconnectivity: Direct links to other relevant state pages, such as Government portals or others. - Search Engine: The website offers a search engine option for user convenience. |
| **Dimension 4: Interaction**  This dimension assesses the effectiveness of communication channels provided by the municipality for citizens to engage with governmental authorities and provide feedback. It encompasses various means of communication and opportunities for citizen participation. |
| - Contact: Direct channels for contacting the municipality (phone, email, etc.). - Platform: Interactive communication channels with users (social networks, online chat, etc.). - Evaluation: Opportunity for users to provide observations, opinions, or suggestions to the municipality. |
| **Dimension 5: Integration**  his dimension evaluates the extent to which the website incorporates inclusive programs and services for marginalized groups, specifically focusing on women and individuals with disabilities. It assesses the website's efforts to provide equitable access and support to these communities. |
| - Inclusion for Women: Section dedicated to services tailored for women. - Disability Inclusion: Section dedicated to services tailored for individuals with disabilities. |
| **Dimension 6: E-democracy**  This dimension evaluates the extent to which the website facilitates citizen participation and engagement in governance processes through online platforms and services. It focuses on the provision of digital tools and resources aimed at fostering democratic practices and citizen empowerment. |
| - Legal Framework: Official laws or decrees governing citizen participation within the municipality. - Activities: Events or initiatives promoting citizen engagement (forums, plebiscites, etc.). - Feedback: Website section enabling the community to share opinions, suggestions, or complaints. - Fund: Neighborhood development fund or other financial resources allocated for citizen participation initiatives. |
| **Dimension 7: Security**  This dimension highlights the municipality's commitment to promoting and maintaining user safety, both online and offline, through proactive measures, resources, and collaboration with relevant stakeholders to address security challenges and protect the well-being of citizens. |
| - Privacy: Indicates if the website has a privacy policy safeguarding user data in the virtual environment, including provisions for victim support. - Security Department: Entity dedicated to managing citizen security concerns. - Programs: Manuals and safety initiatives aimed at enhancing community security. |
