# Supplementary material for: Introducing the municipal digital offering index for evaluating online services and addressing the digital divide
Source: PeerJ Comput Sci. 2025 Sep 3;11:e3049. doi: 10.7717/peerj-cs.3049 (PMC12453795; doi:10.7717/peerj-cs.3049)
Supplement: Supplemental Information 2 [file peerj-cs-11-3049-s002.docx]

**Table A2: Factor Loadings**

| **1.- Municipal Unit and Official Communication** | **Factor Load** | **5.- Lobbying Regulations** | **Factor Load** |
| --- | --- | --- | --- |
| Councilors' Information | 0,53 | Entities Under Lobbying | 0,94 |
| Municipal Institution | 0,79 | Entities Engaging in Lobbying | 0,94 |
| Community Development Department (DIDECO) | 0,93 | Meeting Records | 0,92 |
| Municipal Works | 0,87 | Travel Records | 0,95 |
| Municipal Office | 0,89 | Donations Records | 0,88 |
| Municipal Urban Planning | 0,85 | Lobbying Law Overview | 0,72 |
| Administration Team | 0,49 | **6.- COSOC** | **Factor Load** |
| Transit and Public Transportation | 0,77 | Council Meeting Minutes Access | 0,50 |
| Community Development | 0,84 | Council Description | 0,83 |
| Administrative Services | 0,79 | Council Members | 0,83 |
| Legal Services | 0,76 | Election/Agreement Minutes | 0,88 |
| Environmental Services | 0,76 | Council Regulations | 0,83 |
| **2.-** **Integration Efforts** | **Factor Load** | Council Contact Information | 0,83 |
| Senior Citizen Activities | 0,48 | **7.- Benefits, Responsibilities, and Fees** | **Factor Load** |
| Women´s Inclusion Programs | 0,75 | Community Membership Card | 0,67 |
| Women´s Inclusion Workshops | 0,67 | Neighbor Card Application/Renewal | 0,80 |
| Disability Inclusion Programs | 0,77 | Police Court Fine Payment | 0,67 |
| Disability Inclusion Workshops | 0,65 | Neighbor Card Application Requirements | 0,70 |
| Women´s Support Network | 0,77 | Community Forums | 0,43 |
| Disability Support Network | 0,81 | License Payment | 0,50 |
| Priority Assistance for Disabilities | 0,85 | Fine/Penalty Payment | 0,51 |
| Disability Work Inclusion | 0,80 | Police Court Information | 0,43 |
| Women´s Work Inclusion | 0,75 | **8.- Informative Content Distribution** | **Factor Load** |
| **3.- Transparency Measures** | **Factor Load** | Community Consultations | 0,51 |
| Legal Information | 0,92 | Municipal Referendums | 0,73 |
| Information Requesting | 0,73 | Community Development Fund" | 0,79 |
| Request Tracking | 0,81 | **9.- Community Project Initiatives** | **Factor Load** |
| Complaint Handling | 0,81 | Total Budget | 0,52 |
| Public Financial Reports | 0,82 | Specific Budget | 0,71 |
| Last Update Date | 0,56 | Start Date/End Date | 0,73 |
| Budget Allocation and Execution | 0,68 | **10.- Online Transaction Processes** | **Factor Load** |
| Financial Reports | 0,55 | Certificate Request | 0,50 |
| External Engagements | 0,55 | License Application | 0,88 |
| Vehicle Registration Information | 0,61 | Renewal or Update of Vehicle Registration Permit | 0,66 |
| **4.- Educational and Health Resources** | **Factor Load** | Procedure Inquiry | 0,45 |
| Municipal Schools Directory | 0,80 | Information Regarding Vehicle Registration Permit | 0,67 |
| Children´s Extracurricular Programs | 0,66 | **11.- Ethical standards** | **Factor Load** |
| Adult Education Programs | 0,57 | Mission | 0,94 |
| Education Updates | 0,64 | Vision | 0,90 |
| Education Department Address | 0,76 | Integrity Code | 0,44 |
| Medical Centers Directory | 0,72 | **12.- Citizen Engagement and Consultation** | **Factor Load** |
| Emergency Contacts | 0,80 | Online Chat | 0,50 |
| Healthcare Partnerships | 0,46 | Reports on Citizen Queries | 0,52 |
| Specialty Healthcare Center | 0,46 | **13.- Orientation Services** | **Factor Load** |
| Health Initiatives | 0,53 | Contingent Information | 0,60 |
| Health Updates | 0,69 | Chile Serves | 0,64 |
| Health Department Address | 0,76 | **14.- Environmental Initiatives** | **Factor Load** |
| **15.- Security and Reliability Standards** | **Factor Load** | Recycling Centers / Clean Points | 0,55 |
| Municipal Security Department Address | 0,30 | SCAM | 0,76 |
| Privacy Policies | 0,83 | Environmental Policy | 0,83 |
| Security Manual or Recommendations | 0,47 |  |  |
