# Supplementary material for: Introducing the municipal digital offering index for evaluating online services and addressing the digital divide
Source: PeerJ Comput Sci. 2025 Sep 3;11:e3049. doi: 10.7717/peerj-cs.3049 (PMC12453795; doi:10.7717/peerj-cs.3049)
Supplement: Supplemental Information 7 [file peerj-cs-11-3049-s007.docx]

# Questionnaire (Research Instrument)

**Dimension 1: Municipal Data (17 variables)**

| **Variable** |
| --- |
| Municipality Name |
| Region |
| Community Development Index (2020) |
| Size |
| Population Quantity |
| Poverty Rate |
| Population Density |
| Urban Population (%) |
| No Schooling (%) |
| Basic Education (%) |
| Intermediate Education (%) |
| Higher Education (%) |
| Municipal Personnel Expenses (MM$) |
| Total Municipal Expenses (Accrued in Thousand $) |
| Income from Municipal Patents (MM $) |
| Income from Municipal Circulation Permits (MM $) |
| Income poverty (%) |

**Dimension 2: Community Information Hub (92 variables)**

| **Subdimension** | **Variable** | **Presence in Web Site** |
| --- | --- | --- |
| Information | FAQs | Yes/No |
| Information | Current Events | Yes/No |
| Information | Vaccination Schedule | Yes/No |
| Benefits | Scholarships | Yes/No |
| Benefits | Subsidies | Yes/No |
| Benefits | Community Card | Yes/No |
| Benefits | Public Competitions | Yes/No |
| Municipal Info | Mission | Yes/No |
| Municipal Info | Vision | Yes/No |
| Municipal Info | Community Services Schedule | Yes/No |
| Municipal Structure | Organizational Chart | Yes/No |
| Municipal Structure | Mayor's Information | Yes/No |
| Municipal Structure | Councilors' Information | Yes/No |
| Municipal Structure | Access to Municipal Council Minutes Access | Yes/No |
| COSOC | Description | Yes/No |
| COSOC | Members | Yes/No |
| COSOC | Election Records & Agreements | Yes/No |
| COSOC | Regulations | Yes/No |
| COSOC | Contact | Yes/No |
| Culture and Recreation | Municipal Theater | Yes/No |
| Culture and Recreation | Sports | Yes/No |
| Culture and Recreation | Parks | Yes/No |
| Culture and Recreation | Courses & Workshops (Children's/Youth/Adult) | Yes/No |
| Culture and Recreation | Senior Citizens Activities | Yes/No |
| Culture and Recreation | Municipal Museum & Exhibitions | Yes/No |
| Culture and Recreation | Bike Lanes | Yes/No |
| Education | Municipal Schools List | Yes/No |
| Education | Children’s Extracurricular Programs | Yes/No |
| Education | Adult Education Programs | Yes/No |
| Education | Education News | Yes/No |
| Education | Municipal Education Department Address | Yes/No |
| Health | Medical Centers List | Yes/No |
| Health | Emergency & Urgent Care Hotlines | Yes/No |
| Health | Health Agreements | Yes/No |
| Health | Community Pharmacy | Yes/No |
| Health | Municipal Specialty Center | Yes/No |
| Health | Health News | Yes/No |
| Health | Municipal Health Department Address | Yes/No |
| Environment | Recycling Centers & Drop-Off Points | Yes/No |
| Environment | SCAM (Municipal Environmental Certification System) | Yes/No |
| Environment | Environmental Policy | Yes/No |
| Municipal Units | Municipal Institution | Yes/No |
| Municipal Units | Community Education Directorate & Department (DIDECO) | Yes/No |
| Municipal Units | Municipal Offices | Yes/No |
| Municipal Units | Municipal Secretariat | Yes/No |
| Municipal Units | Communal Planning Secretariat | Yes/No |
| Municipal Units | Executive Cabinet | Yes/No |
| Municipal Units | Environment, Sanitation & Beautification | Yes/No |
| Municipal Units | Traffic & Public Transport | Yes/No |
| Municipal Units | Community Development | Yes/No |
| Municipal Units | Municipal Administration | Yes/No |
| Municipal Units | Legal Advisory | Yes/No |
| Projects | Total Budget | Yes/No |
| Projects | Allocated Budget | Yes/No |
| Projects | Project Description | Yes/No |
| Projects | Start & End Date | Yes/No |
| Projects | Contracted Company | Yes/No |
| Transparency Law | Municipal Policies and Legal Regulations | Yes/No |
| Transparency Law | Reports on Activities of Neighborhood Councils and Community Organizations, including Electoral Processes | Yes/No |
| Transparency Law | Municipal Costs and General Cost History | Yes/No |
| Transparency Law | Public Bidding Processes | Yes/No |
| Transparency Law | Regulatory Plan | Yes/No |
| Transparency Law | Normative Framework | Yes/No |
| Transparency Law | Declaration of Assets and Interests | Yes/No |
| Transparency Law | Transfers of Funds and Economic Contributions Delivered | Yes/No |
| Transparency Law | General Information on the Law | Yes/No |
| Transparency Law | Information Request Submission Portal | Yes/No |
| Transparency Law | Tracking of Requests | Yes/No |
| Transparency Law | Complaints | Yes/No |
| Transparency Law | Public Accountability Report | Yes/No |
| Transparency Law | Date of Last Website Update | Yes/No |
| Transparency Law | Acts and Documents Published in the Official Gazette | Yes/No |
| Transparency Law | Legal Powers and Authorities | Yes/No |
| Transparency Law | Organizational Structure | Yes/No |
| Transparency Law | Internal Organization Regulations | Yes/No |
| Transparency Law | Employee Remunerations | Yes/No |
| Transparency Law | Procurement and Contracting | Yes/No |
| Transparency Law | Code of Integrity | Yes/No |
| Transparency Law | Acts and Resolutions Affecting Third Parties | Yes/No |
| Transparency Law | Assigned Budgets and Their Execution | Yes/No |
| Transparency Law | Financial Status | Yes/No |
| Transparency Law | Audits | Yes/No |
| Transparency Law | Participation in Other Entities | Yes/No |
| Transparency Law | Annual Report to SUBDERE (Subsecretariat for Regional and Administrative Development) | Yes/No |
| Transparency Law | Technical Guidelines | Yes/No |
| Lobbying Law | Passive Entities | Yes/No |
| Lobbying Law | Active Entities | Yes/No |
| Lobbying Law | Meetings and Hearings | Yes/No |
| Lobbying Law | Travel Expenses | Yes/No |
| Lobbying Law | Donations | Yes/No |
| Lobbying Law | Information on the Lobby Law | Yes/No |

**Dimension 3: Bidirectional Transactions (26)**

| **Subdimension** | **Variable** | **Presence in Web Site** |
| --- | --- | --- |
| Online Processing | Patent Payment | Yes/No |
| Online Processing | Workshop Registration / Community Development Programs | Yes/No |
| Online Processing | Fine Payment / Ticket Processing | Yes/No |
| Online Processing | Vehicle Permit Renewal | Yes/No |
| Online Processing | Neighborhood Card Issuance or Renewal | Yes/No |
| Online Processing | Local Police Court Fine Payment | Yes/No |
| Online Processing | Certificate Requests | Yes/No |
| Online Processing | Patent Application | Yes/No |
| Online Processing | Vehicle Permit Renewal or Update Request | Yes/No |
| Online Processing | Ownership Transfer Request | Yes/No |
| Online Processing | Household Waste Collection Payment | Yes/No |
| Online Processing | Procedure Inquiry | Yes/No |
| Online Processing | Form Downloads | Yes/No |
| Information on Procedures | Fine Information | Yes/No |
| Information on Procedures | Vehicle Permit Regulations | Yes/No |
| Information on Procedures | Requirements for Obtaining a Neighborhood Card | Yes/No |
| Information on Procedures | Local Police Court Locations | Yes/No |
| Information on Procedures | Social Registry of Households Information | Yes/No |
| Information on Procedures | Fine Information | Yes/No |
| Information on Procedures | Vehicle Permit Regulations | Yes/No |
| Online Signature | Electronic Signature | Yes/No |
| Online Signature | Unique Key Signature | Yes/No |
| Interconnectivity | Chile Atiende (Government Service Portal) | Yes/No |
| Interconnectivity | Official Government Website | Yes/No |
| Interconnectivity | Association of Municipalities | Yes/No |
| Search Engine | Search engine | Yes/No |

**Dimension 4: Interaction (5 variables)**

| **Subdimension** | **Variable** | **Presence in Web Site** |
| --- | --- | --- |
| Contact | Contact Number | Yes/No |
| Contact | Email Addresses | Yes/No |
| Platform | Online Chat | Yes/No |
| Platform | Social Media | Yes/No |
| Evaluation | Service Quality Evaluation Systems | Yes/No |

**Dimension 5: Integration (9 variables)**

| **Subdimension** | **Variable** | **Presence in Web Site** |
| --- | --- | --- |
| Women inclusion | Women's Inclusion Programs | Yes/No |
| Women inclusion | Women's Inclusion Workshops | Yes/No |
| Disability Inclusion | Disability Inclusion Programs | Yes/No |
| Disability Inclusion | Disability Inclusion Workshops | Yes/No |
| Women inclusion | Women's Support Network | Yes/No |
| Disability Inclusion | Disability Support Network | Yes/No |
| Disability Inclusion | Priority Assistance for People with Disabilities | Yes/No |
| Disability Inclusion | Labor Inclusion for People with Disabilities | Yes/No |
| Women inclusion | Labor Inclusion for Women | Yes/No |

**Dimension 6: E-Democracy (8 variables)**

| **Subdimension** | **Variable** | **Presence in Web Site** |
| --- | --- | --- |
| Legal Framework | General Participation Guidelines | Yes/No |
| Activities | Public Hearings | Yes/No |
| Activities | Municipal Plebiscites | Yes/No |
| Feedback | Complaints, Presentations, Citizen Opinions, Suggestions & Information | Yes/No |
| Activities | Community Organizations | Yes/No |
| Fund | Neighborhood Development Fund | Yes/No |
| Activities | Municipal Forums | Yes/No |
| Feedback | Reports on Citizen Consultations | Yes/No |

**Dimension 7: Security (6 variables)**

| **Subdimension** | **Variable** | **Presence in Web Site** |
| --- | --- | --- |
| Privacy | Privacy Policies | Yes/No |
| Security Department | Municipal Security Department Address | Yes/No |
| Security Department | Citizen Security Number | Yes/No |
| Programs | Security Manual & Recommendations | Yes/No |
| Programs | Security Programs | Yes/No |
| Security Department | Victim Assistance | Yes/No |
