# Supplementary material for: Introducing the municipal digital offering index for evaluating online services and addressing the digital divide
Source: PeerJ Comput Sci. 2025 Sep 3;11:e3049. doi: 10.7717/peerj-cs.3049 (PMC12453795; doi:10.7717/peerj-cs.3049)
Supplement: Supplemental Information 8 [file peerj-cs-11-3049-s008.docx]

# Cuestionario (Instrumento de Investigación) Dimensión 1: Datos Municipales (17 variables)

| **Variable** |
| --- |
| Nombre del Municipio |
| Región |
| Índice de Desarrollo Comunitario (2020) |
| Tamaño |
| Población |
| Tasa de Pobreza |
| Densidad Poblacional |
| Población Urbana (%) |
| Sin Escolarización (%) |
| Educación Básica (%) |
| Educación Intermedia (%) |
| Educación Superior (%) |
| Gastos en Personal Municipal (MM$) |
| Gastos Totales Municipales (Devengados en Mil $) |
| Ingresos por Patentes Municipales (MM$) |
| Ingresos por Permisos de Circulación Municipales (MM$) |
| Pobreza por Ingresos (%) |

**Dimensión 2: Centro de Información Comunitaria (92 variables)**

| **Subdimensión** | **Variable** | **Presencia en sitio Web** |
| --- | --- | --- |
| Información | Preguntas frecuentes | Sí/No |
| Información | Actualidades | Sí/No |
| Información | Calendario de vacunación | Sí/No |
| Beneficios | Becas | Sí/No |
| Beneficios | Subsidios | Sí/No |
| Beneficios | Tarjeta de la Comunidad | Sí/No |
| Beneficios | Licitaciones Públicas | Sí/No |
| Info Municipal | Misión | Sí/No |
| Info Municipal | Visión | Sí/No |
| Información Municipal | Horario de Servicios Comunitarios | Sí/No |
| Estructura Municipal | Organigrama | Sí/No |
| Estructura Municipal | Información del Alcalde | Sí/No |
| Estructura Municipal | Información de los concejales | Sí/No |
| Estructura Municipal | Acceso a las Actas del Concejo Municipal | Sí/No |
| COSOC | Descripción | Sí/No |
| COSOC | Miembros | Sí/No |
| COSOC | Registros Electorales y Acuerdos | Sí/No |
| COSOC | Normativa | Sí/No |
| COSOC | Contacto | Sí/No |
| Cultura y Recreación | Teatro Municipal | Sí/No |
| Cultura y Recreación | Deportes | Sí/No |
| Cultura y Recreación | Parques | Sí/No |
| Cultura y Recreación | Cursos y Talleres (Niños/Jóvenes/Adultos) | Sí/No |
| Cultura y Recreación | Actividades para Personas Mayores | Sí/No |
| Cultura y Recreación | Museo Municipal y Exposiciones | Sí/No |
| Cultura y Recreación | Ciclovías | Sí/No |
| Educación | Lista de Escuelas Municipales | Sí/No |
| Educación | Programas Extracurriculares para Niños | Sí/No |
| Educación | Programas de Educación para Adultos | Sí/No |
| Educación | Noticias de Educación | Sí/No |
| Educación | Dirección del Departamento Municipal de Educación | Sí/No |
| Salud | Lista de Centros Médicos | Sí/No |
| Salud | Líneas directas de atención de emergencia y urgencia | Sí/No |
| Salud | Acuerdos de Salud | Sí/No |
| Salud | Farmacia Comunitaria | Sí/No |
| Salud | Centro Municipal de Especialidades | Sí/No |
| Salud | Noticias de Salud | Sí/No |
| Salud | Dirección del Departamento Municipal de Salud | Sí/No |
| Medio Ambiente | Centros de reciclaje y puntos de entrega | Sí/No |
| Medio Ambiente | SCAM (Sistema de Certificación Ambiental Municipal) | Sí/No |
| Medio Ambiente | Política Ambiental | Sí/No |
| Unidades Municipales | Institución Municipal | Sí/No |
| Unidades Municipales | Dirección y Departamento de Educación Comunitaria (DIDECO) | Sí/No |
| Unidades Municipales | Oficinas Municipales | Sí/No |
| Unidades Municipales | Secretaría Municipal | Sí/No |
| Unidades Municipales | Secretaría de Planificación Comunal | Sí/No |
| Unidades Municipales | Gabinete Ejecutivo | Sí/No |
| Unidades Municipales | Medio Ambiente, Saneamiento y Embellecimiento | Sí/No |
| Unidades Municipales | Tráfico y transporte público | Sí/No |
| Unidades Municipales | Desarrollo Comunitario | Sí/No |
| Unidades Municipales | Administración Municipal | Sí/No |
| Unidades Municipales | Asesoría Legal | Sí/No |
| Proyectos | Presupuesto total | Sí/No |
| Proyectos | Presupuesto asignado | Sí/No |
| Proyectos | Descripción del proyecto | Sí/No |
| Proyectos | Fecha de inicio y finalización | Sí/No |
| Proyectos | Empresa contratada | Sí/No |
| Ley de Transparencia | Políticas Municipales y Regulaciones Legales | Sí/No |
| Ley de Transparencia | Informes sobre las actividades de las juntas vecinales y las organizaciones comunitarias, incluidos los procesos electorales | Sí/No |
| Ley de Transparencia | Costos Municipales e Historial General de Costos | Sí/No |
| Ley de Transparencia | Procesos de Licitación Pública | Sí/No |
| Ley de Transparencia | Plan Regulatorio | Sí/No |
| Ley de Transparencia | Marco normativo | Sí/No |
| Ley de Transparencia | Declaración de Bienes e Intereses | Sí/No |
| Ley de Transparencia | Transferencias de fondos y contribuciones económicas entregadas | Sí/No |
| Ley de Transparencia | Información general sobre la ley | Sí/No |
| Ley de Transparencia | Portal de Presentación de Solicitudes de Información | Sí/No |
| Ley de Transparencia | Seguimiento de solicitudes | Sí/No |
| Ley de Transparencia | Quejas | Sí/No |
| Ley de Transparencia | Informe de Responsabilidad Pública | Sí/No |
| Ley de Transparencia | Fecha de la última actualización del sitio web | Sí/No |
| Ley de Transparencia | Actos y documentos publicados en el Diario Oficial | Sí/No |
| Ley de Transparencia | Poderes y Autoridades Legales | Sí/No |
| Ley de Transparencia | Estructura Organizacional | Sí/No |
| Ley de Transparencia | Reglamento Interno de Organización | Sí/No |
| Ley de Transparencia | Remuneraciones de los empleados | Sí/No |
| Ley de Transparencia | Adquisiciones y Contrataciones | Sí/No |
| Ley de Transparencia | Código de Integridad | Sí/No |
| Ley de Transparencia | Actos y Resoluciones que Afectan a Terceros | Sí/No |
| Ley de Transparencia | Presupuestos asignados y su ejecución | Sí/No |
| Ley de Transparencia | Estado financiero | Sí/No |
| Ley de Transparencia | Auditorías | Sí/No |
| Ley de Transparencia | Participación en otras entidades | Sí/No |
| Ley de Transparencia | Informe Anual a la SUBDERE (Subsecretaría de Desarrollo Regional y Administrativo) | Sí/No |
| Ley de Transparencia | Lineamientos Técnicos | Sí/No |
| Ley de Lobby | Entidades pasivas | Sí/No |
| Ley de Lobby | Entidades activas | Sí/No |
| Ley de Lobby | Reuniones y Audiencias | Sí/No |
| Ley de Lobby | Gastos de viaje | Sí/No |
| Ley de Lobby | Donaciones | Sí/No |
| Ley de Lobby | Información sobre la Ley de Lobby | Sí/No |

**Dimensión 3: Tansacciones Bidireccionales (26)**

| **Subdimensión** | **Variable** | **Presencia en sitio Web** |
| --- | --- | --- |
| Procesamiento en Línea | Pago de patentes | Sí/No |
| Procesamiento en Línea | Inscripción a Talleres / Programas de Desarrollo Comunitario | Sí/No |
| Procesamiento en Línea | Pago de multas / procesamiento de multas | Sí/No |
| Procesamiento en Línea | Renovación de Permiso de Vehículo | Sí/No |
| Procesamiento en Línea | Emisión o Renovación de Tarjeta de Vecindario | Sí/No |
| Procesamiento en Línea | Pago de la multa del Tribunal de Policía Local | Sí/No |
| Procesamiento en Línea | Solicitudes de certificados | Sí/No |
| Procesamiento en Línea | Solicitud de patente | Sí/No |
| Procesamiento en Línea | Solicitud de Renovación o Actualización de Permiso de Vehículo | Sí/No |
| Procesamiento en Línea | Solicitud de Transferencia de Propiedad | Sí/No |
| Procesamiento en Línea | Pago de la recogida de residuos domésticos | Sí/No |
| Procesamiento en Línea | Procedimiento de indagación | Sí/No |
| Procesamiento en Línea | Descargas de formularios | Sí/No |
| Información sobre Procedimientos | Información de multa | Sí/No |
| Información sobre Procedimientos | Reglamento de Permisos de Vehículos | Sí/No |
| Información sobre Procedimientos | Requisitos para obtener una tarjeta de vecindario | Sí/No |
| Información sobre Procedimientos | Ubicaciones de los Tribunales de Policía Local | Sí/No |
| Información sobre Procedimientos | Información del Registro Social de Hogares | Sí/No |
| Información sobre Procedimientos | Información de multa | Sí/No |
| Información sobre Procedimientos | Reglamento de Permisos de Vehículos | Sí/No |
| Firma electrónica | Firma electrónica | Sí/No |
| Firma electrónica | Armadura de clave única | Sí/No |
| Interconectivitdad | Chile Atiende (Portal de Servicios Gubernamentales) | Sí/No |
| Interconectivitdad | Sitio web oficial del gobierno | Sí/No |
| Interconectivitdad | Mancomunidad de Municipios | Sí/No |
| Motor de Búsqueda | Motor de búsqueda | Sí/No |

**Dimensión 4: Interacción (5 variables)**

| **Subdimensión** | **Variable** | **Presencia en Sitio Web** |
| --- | --- | --- |
| Contacto | Número de contacto | Sí/No |
| Contacto | Direcciones de correo electrónico | Sí/No |
| Plataforma | Chat en línea | Sí/No |
| Plataforma | Redes Sociales | Sí/No |
| Evaluación | Sistemas de Evaluación de la Calidad del Servicio | Sí/No |

**Dimensión 5: Integración (9 variables)**

| **Subdimensión** | **Variable** | **Presencia en Sitio Web** |
| --- | --- | --- |
| Inclusión de la mujer | Programas de Inclusión de la Mujer | Sí/No |
| Inclusión de la mujer | Talleres de Inclusión de Mujeres | Sí/No |
| Inclusión de la Discapacidad | Programas de Inclusión de Personas con Discapacidad | Sí/No |
| Inclusión de la Discapacidad | Talleres de Inclusión de Personas con Discapacidad | Sí/No |
| Inclusión de la mujer | Red de apoyo a las mujeres | Sí/No |
| Inclusión de la Discapacidad | Red de Apoyo a la Discapacidad | Sí/No |
| Inclusión de la Discapacidad | Asistencia Prioritaria para Personas con Discapacidad | Sí/No |
| Inclusión de la Discapacidad | Inclusión Laboral para Personas con Discapacidad | Sí/No |
| Inclusión de la mujer | Inclusión laboral de las mujeres | Sí/No |

**Dimensión 6: E-Democracia (8 variables)**

| **Subdimensión** | **Variable** | **Presencia en Sitio Web** |
| --- | --- | --- |
| Marco Legal | Lineamientos Generales de Participación | Sí/No |
| Actividades | Audiencias Públicas | Sí/No |
| Actividades | Plebiscitos Municipales | Sí/No |
| Retroalimentación | Quejas, Presentaciones, Opiniones Ciudadanas, Sugerencias e Información | Sí/No |
| Actividades | Organizaciones Comunitarias | Sí/No |
| Fondos | Fondo de Desarrollo Vecinal | Sí/No |
| Actividades | Foros Municipales | Sí/No |
| Retroalimentación | Informes de Consultas Ciudadanas | Sí/No |

**Dimensión 7: Seguridad (6 variables)**

| **Subdimensión** | **Variable** | **Presencia en Sitio Web** |
| --- | --- | --- |
| Privacidad | Políticas de privacidad | Sí/No |
| Departamento de Seguridad | Dirección del Departamento de Seguridad Municipal | Sí/No |
| Departamento de Seguridad | Número de Seguridad Ciudadana | Sí/No |
| Programas | Manual de seguridad y recomendaciones | Sí/No |
| Programas | Programas de seguridad | Sí/No |
| Departamento de Seguridad | Asistencia a las víctimas | Sí/No |
